# Embryonic lethality in mice lacking Trim59 due to impaired gastrulation development

**DOI:** 10.1038/s41419-018-0370-y

**Published:** 2018-02-21

**Authors:** Xiaomin Su, Chenglei Wu, Xiaoying Ye, Ming Zeng, Zhujun Zhang, Yongzhe Che, Yuan Zhang, Lin Liu, Yushuang Lin, Rongcun Yang

**Affiliations:** 1 Department of Immunology Nankai University School of Medicine, Nankai University, Tianjin, China; 2 Key Laboratory of Bioactive Materials Ministry of Education, Nankai University, Tianjin, China; 3 State Key Laboratory of Medicinal Chemical Biology, Nankai University, Tianjin, China; 4 College of Life Sciences, Nankai University, Tianjin, China; 5 Shandong Provincial Key Laboratory of Animal Cells and Developmental Biology, School of Life Sciences, Shandong University, Shandong, 250100 China

## Abstract

TRIM family members have been implicated in a variety of biological processes such as differentiation and development. We here found that Trim59 plays a critical role in early embryo development from blastocyst stage to gastrula. There existed delayed development and empty yolk sacs from embryonic day (E) 8.5 in *Trim59−/−* embryos. No viable *Trim59−/−* embryos were observed beyond E9.5. Trim59 deficiency affected primary germ layer formation at the beginning of gastrulation. At E6.5 and E7.5, the expression of primary germ layer formation-associated genes including *Brachyury*, *lefty2*, *Cer1*, *Otx2*, *Wnt3,* and *BMP4* was reduced in *Trim59−/−* embryos. Homozygous mutant embryonic epiblasts were contracted and the mesoderm was absent. Trim59 could interact with actin- and myosin-associated proteins. Its deficiency disturbed F-actin polymerization during inner cell mass differentiation. Trim59-mediated polymerization of F-actin was via WASH K63-linked ubiquitination. Thus, Trim59 may be a critical regulator for early embryo development from blastocyst stage to gastrula through modulating F-actin assembly.

## Introduction

Mouse embryological development is a dynamic process. It includes a series of cleavage divisions during the development from embryonic day (E) 0.5 to E8.0^1^. Following fertilization, one cell embryo (E0.5) undertakes a succession of cleavage divisions to generate a blastocyst (E4.5) that contains inner cell mass (ICM) and trophectoderm (TE). The ICM contains epiblast (EPI) precursor and primitive endoderm (PrE). From E5.5 to E6.0, pre-gastrula is formed during this stage. The appearance of anterior visceral endoderm (AVE) represents a crucial event in the patterning of anterior–posterior (A–P) axis, and formation of the primitive steak (PS) marks the beginning of gastrulation at E6.5. During this stage, the primary germ layers including ectoderm (EC), mesoderm (ME), and endoderm (EN) are formed^2^.

During gastrulation, PS formation requires interactions between EPI, extra-embryonic AVE, and extra-embryonic ectoderm (ExE). Multiple genes and signaling pathways are involved in the regulation of embryogenesis. In mouse embryos, EPI expresses *Nodal*, whereas ExE expresses bone morphogenetic protein 4 (*BMP4*). These molecules are involved in many steps in pregastrulation development. With the growth of EPI, some of AVE genes are expressed in distal visceral endoderm (DVE) cells at E5.5. *Otx2* (Orthodenticle homeobox 2), *Cer1* (Cerberus 1), and *Lefty2* belong to AVE genes^3^. *Otx2* is a transcription factor, whereas *Cer1* and *Lefty2* belong to signaling antagonists^4^. Signaling pathways such as *β-catenin*/*Wnt3*, *Nodal*, *BMP4*, and *FG*F are considered as essential to AVE formation^5^. *Nodal* and *Wnt3* are expressed in the proximal epiblast adjacent to the extraembryonic ectoderm, next to where the PS arises, whereas *BMP4* is expressed in the distal ExE^6^. At the same time, other posterior genes such as *Brachyury* and *Cripto* also play a critical role in the formation of primitive streak (PS) and definitive endoderm (DE)^7^.

Mouse embryological development is a complex developmental program with correcting mechanisms to avoid the transmission of errors^8^. However, the mechanism(s) of maintaining normal mouse embryological development is not completely understood. Multiple processes such as cell–cell contact, gene expression, cell signaling pathways, positional relationships and epigenetics, control cell lineage specification from blastocyst to three germ layer formation. However, actin remodeling may also be important for normal embryo development. Studies have shown that cell actin skeleton is regulated by many proteins that either promote or inhibit actin polymerization during embryo development^9^.

In this study, we investigate the impact of Trim59 on the early embryonic development. Trim59, a member of the tripartite motif (TRIM)-containing protein superfamily, is characterized by one or two zinc binding motifs, an associated coiled-coil region and a RING-finger domain^10^. Previous studies show that Trim59 participates in many pathological regulation such as inflammation^11^, cytotoxicity^12^, and especially tumorigenesis^13^. Here we found that Trim59 plays a vital role in mouse early embryonic development stage. Trim59 may promote F-actin assembly through WASH K63-linked ubiquitination during blastocyst develops into gastrula stage. Trim59 deficiency affects the formation of primary germ layers ectoderm, mesoderm, and endoderm.

## Results

### Trim59 deficiency causes early embryonic lethality

Trim59 could be detected not only in murine F1 ESCs (embryonic stem cells derived from C57BL/6 × C3H F1 mouse) but also in wild-type (wt) murine E6.5–E9.5 embryos (Supplementary Figure S1), implying that Trim59 may play a role in embryonic development. To test this, we generated *Trim59* knockout (*−/−*) mice (Supplementary Figure S2). We observed more than six generations within a year and found that around 67% of offsprings (70 animals) were *Trim59*+*/−*, and 33% (34 animals) were *Trim59*+/+, whereas no offspring was *Trim59−/−* (Table 1), indicating that *Trim59−/−* genotype is embryonic lethality. To determine the stage of embryonic lethality, *Trim59*+*/−* mice were crossed and then embryos were dissected for genotyping. At E6.5, 12 (29%) were *Trim59*+/+, 20 (49%) *Trim59*+*/−*, and 9 (22%) *Trim59−/−* among 41 decidua examined, fitting well to a single-gene inheritance model. These data suggest that *Trim59* knockout does not affect the implantation and decidualization. However, the development degree of *Trim59−/−* embryos was delayed at this stage (Table 1). Similar phenomenon was also observed at E7.5 (Table 1). At E8.5, some empty swollen decidua embryos could be observed in the dissected embryos. These abnormal embryos were *Trim59−/−* genotype (Table 1). Of the 57 offspring examined, 20 (35%) were empty, 11 (19%) contained *Trim59*+/+ embryos, and 26 (46%) were *Trim59*+*/−* embryos (Table 1). No *Trim59−/−* embryos were detected at E9.5 (Table 1). Taken together, these results indicate that Trim59 is essential for early embryonic development.

**Table 1:** Genotype analyses of offsprings from Trim59+/− intercross

Stage	+/+	+/−	−/−	Total	Phenotype (-/-)
E6.5	12	20	9	41	Delayed
E7.5	10	29	11	50	Delayed
E8.5	13	24	12	49	Empty yolk sacs
E9.5	11	26	0 (20*)	57	Resorbent
Postnatal	34	70	0	104	

Numbers indicate born mice or embryos detected at different stages of gestation

### Trim59 deficiency affects gastrulation during early embryonic development

**Fig. 1:**
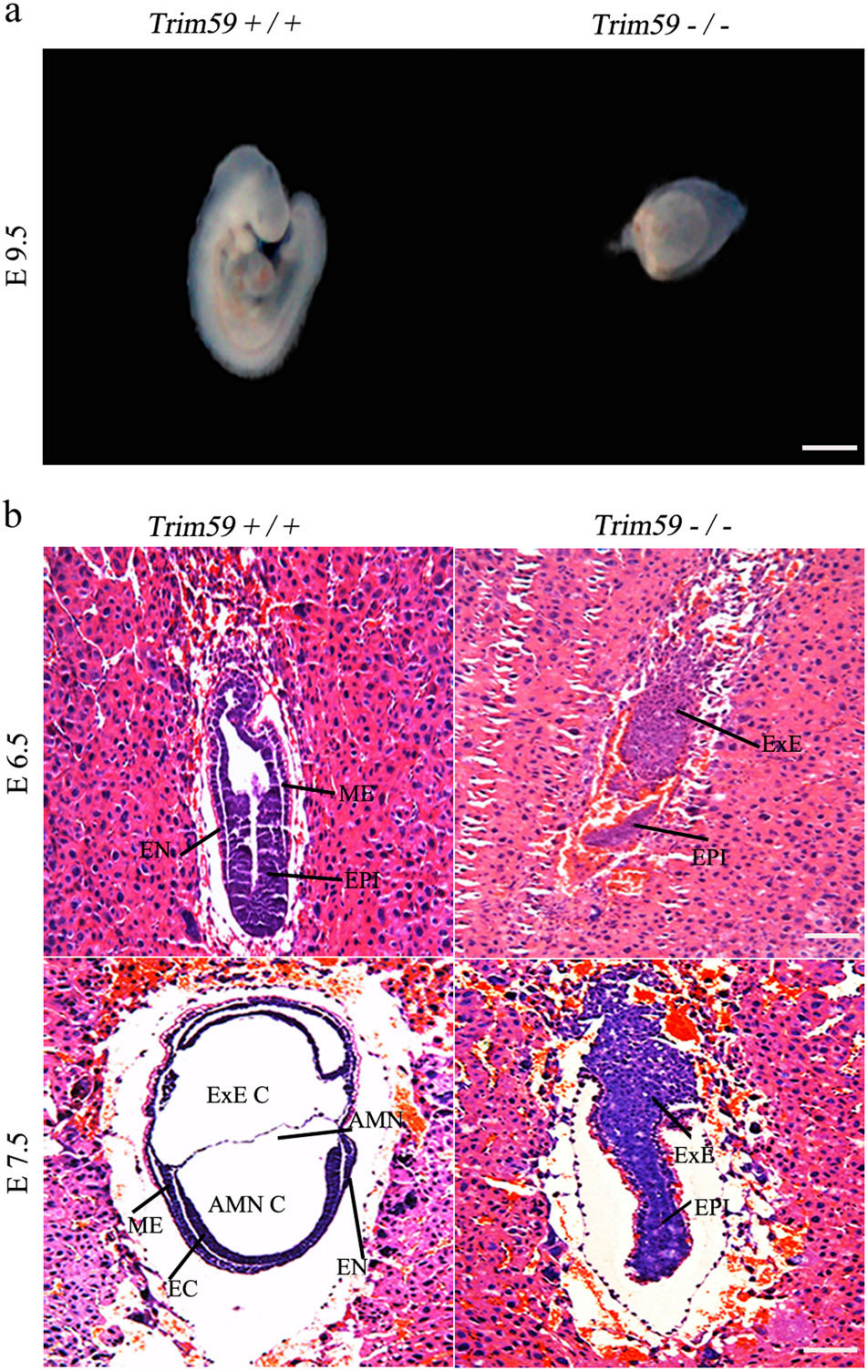
Trim59 is essential for normal embryogenesis. **a** Wild-type (*Trim59*+/+) and Trim59 knockout mice (*Trim59−/−*) embryos. Representative images of foetuses at E9.5 were displayed. **b** H&E staining of *Trim59*+/+and *Trim59−/−* embryos at E6.5 and E7.5. Mesoderm (ME), entoderm (EN), ectoderm (EC), epiblast (EPI), extra-embryonic ectoderm (ExE), extra-embryonic coelom (ExE C), amnion (AMN), amnion cavity (AMN C). Scale bar, 100 μm

Due to some empty swollen decidua embryos were detected in the dissected embryos at E9.5 (Fig. 1a), Trim59 may affect gastrulation that initiates from E6.5 and end at E7.5. Hematoxylin/eosin (H&E) staining revealed that *Trim59*+/+ embryos exhibited normal morphology with the remodeling of epiblast, the formation of epithelium around a central lumen, which leads to the formation of postimplantation epiblast, and the emergence of primitive streak and mesoderm at E6.5 (Fig. 1b). At E7.5, the embryos develop into the late stage of gastrulation. During this stage, aminion, aminion cavity, ExE cavity, and intact three germ layers were observed in wt embryos, whereas similar structure was not found in *Trim59−/−* embryos. Conversely, contracted embryonic epiblast, absent mesoderm and collapsed skeleton often appeared in *Trim59−/−* embryos at E6.5 (Fig. 1b). At E7.5, yolk sac and amnion were also defective in *Trim59−/−* embryos and no normal three germ layers were observed (Fig. 1b). These results suggest that Trim59 knockout affects gastrulation during early embryos development.

**Fig. 2:**
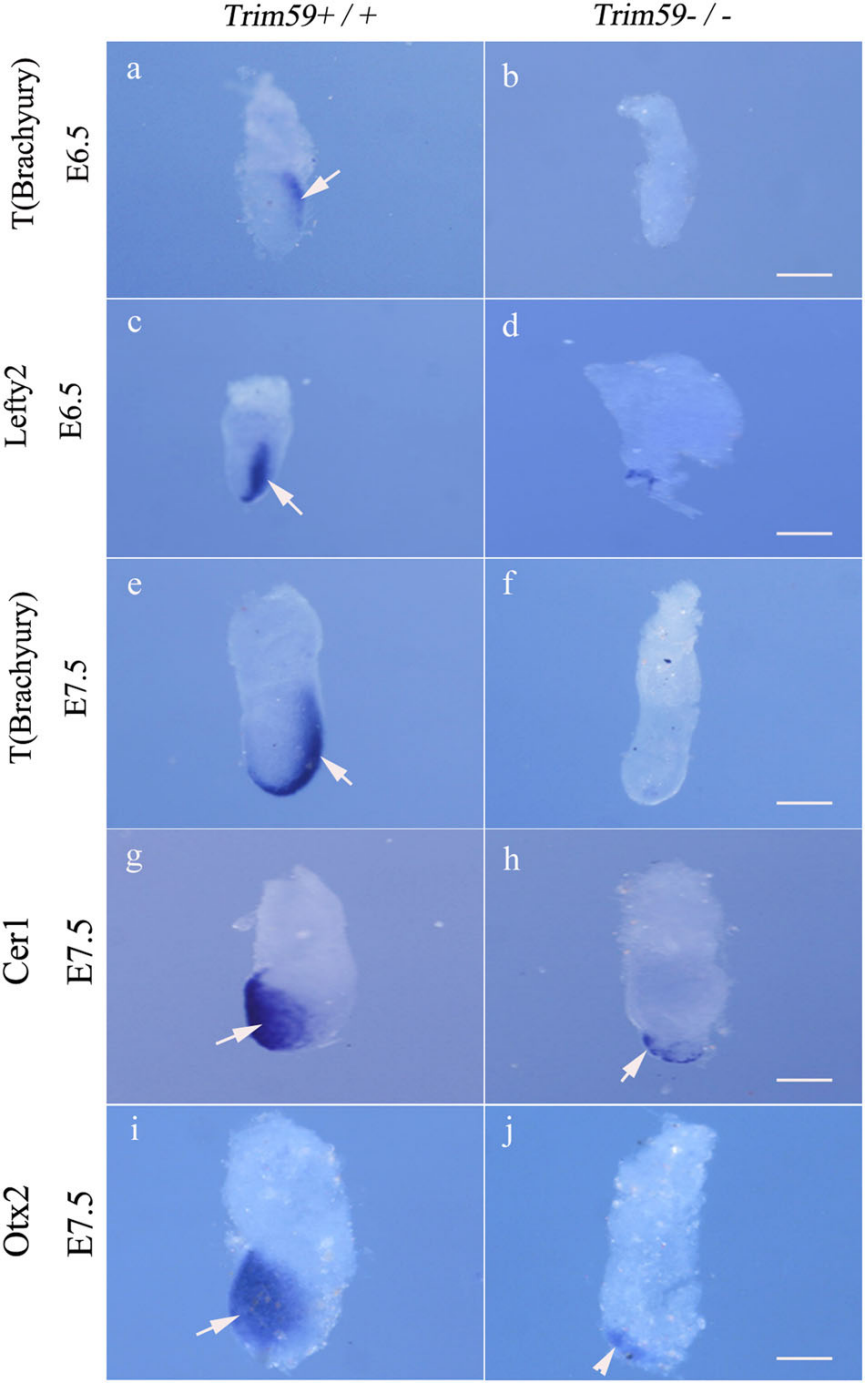
Trim59 deficiency affects expression of gastrulation-associated genes. Embryos at E6.5 were hybridization using a digoxigenin-labeled anti-sense RNA probe to mouse *T (Brachyury)* (**a**, **b**) and *Lefty2* (**c**, **d**). Embryos at E7.5 were hybridization using a digoxigenin-labeled anti-sense RNA probe to mouse *Brachyury* (**e**, **f**), *Cer1* (**g**, **h**), and *Otx2* (**i**, **j**). Scale bar in all panels indicates 100 μm. Arrow indicates *Brachyury*, *Lefty2*, *Cer1*, or *Otx2*

Next, we further characterized lethal stage of the early embryonic development in Trim59 knockout mice. Formation of PS happens around E6.5, which originates from an elongated thickening of epiblast and marks the beginning of gastrulation^14^. Many molecular markers are involved in the formation of PS including *Brachyury*, *Lefty2*, *Cer1*, and *Otx2* such as that *Brachyury* marks the formation of PS and axial mesoderm. We found that *Brachyury* could not be detected in *Trim59−/−* embryos (Fig. 2a, b). To exclude the possibility of a simple delay in the expression of *Brachyury*, we also tested its expression at E6.5 and E7.5. At neither stages did *Trim59−/−* embryos express *Brachyury* (Fig. 2a, b, e, f), indicating that Trim59 deficiency fails to form PS. *Lefty2*, as a nascent mesoderm marker could be detected at E6.5 in *Trim59*+/+ embryos, whereas *Trim59−/−* embryos were negative (Fig. 2c, d). All of these suggest that Trim59 is necessary for PS formation, which happens at the early gastrulation. During gastrulation, Otx2 in the anterior neuroectoderm (ANE) and Cer1 in the definitive endoderm (DEE) also markedly decreased in Trim59 knockout mice than in wt mice at E7.5 (Fig. 2g–j), representing failed development of ANE and DEE when Trim59 losses its function. These data support our findings that Trim59 is involved in gastrulation during early embryonic development.

**Fig. 3:**
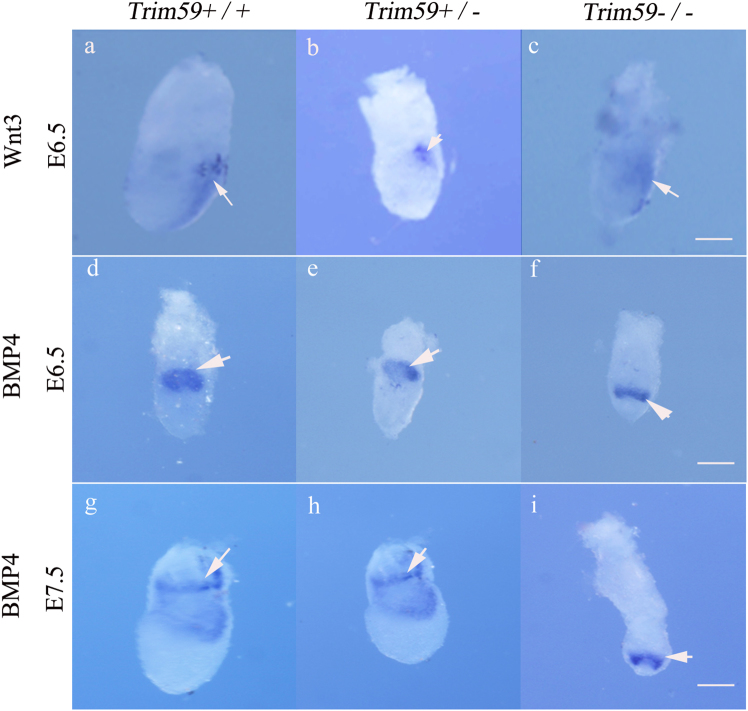
Trim59 deficiency affects Wnt3 and BMP4 expression in gastrulation stage. Embryos were hybridization using a digoxigenin-labeled anti-sense RNA probes to mouse Wnt3 (E6.5) (**a**–**c**); BMP4 (E6.5) (**d**–**f**), and (E7.5) (**g**–**i**). Arrow indicates Wnt3 or BMP4. Scale bar, 100 μm

PS induction depends on the crosstalk between Epi and two extra-embryonic tissues AVE and ExE. During gastrulation, signaling molecules such as *Wnt3*, *BMP4*, *Nodal* and *FGF* are involved in PS formation. At E6.5, *Wnt3* expression was remarkably reduced in the EPI of *Trim59−/−* embryos (Fig. 3a–c). The areas of expression *BMP4* in EPI and ExE were also smaller in size at both E6.5 and E7.5 than in the wt embryos (Fig. 3d–f, g–i). Furthermore, the expression region of *BMP4* moved to EPI at this stage (Fig. 3d–f, g–i). These data show that Trim59 knockout also causes abnormal expression of signaling molecules. Taken together, Trim59 deficiency may cause the failure of gastrulation and affect the expression of gastrulation associated genes and signaling molecules.

### Trim59 deficiency interrupts differentiation of blastocyst inner cell mass

**Fig. 4:**
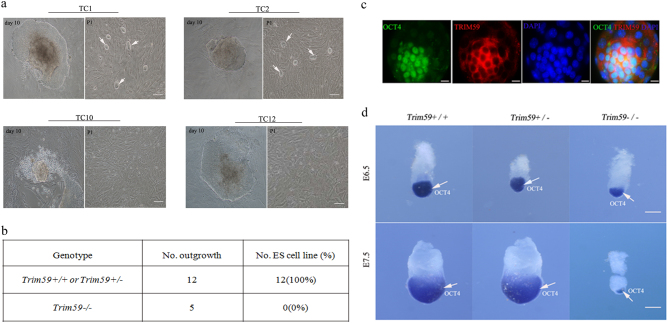
Trim59 deficiency affects differentiation of blastocyst inner cell mass. **a** ES cell clones at passage 1. TC1, TC2, TC10, and TC12, name of outgrowths. TC1 and TC2 were representative images of *Trim59*+/+ or *Trim59*+/*−* genotype, whereas TC10 and TC12 were representative images of *Trim59−/−* genotype. P1 passage 1. Arrows indicate ES cell clones. Scale bar, 10 µm. **b** Growth and differentiation of ES cells from *Trim59*+/+, *Trim59*+*/−* and *Trim59−/−* mice. No. outgrowth number of outgrowths; No. ES cell line number of ES cell line. **c** Staining of Trim59 and Oct4 in F1 ESCs. F1 ESCs was stained by anti-mouse Trim59 and anti-mouse Oct4 antibody. Images were acquired using a Bio-Rad Radiance 2100 confocal microscope with a Zeiss 63× oil immersion objective. Scale bar, 10 µm. **d** Hybridization of Oct4 in embryos. Embryos at E6.5 and E7.5 were hybridization using a digoxigenin-labeled anti-sense RNA probe to mouse Oct4. Scale bar, 100 μm. Arrow indicates Oct4

The stem cells from ICM of blastocyst stage embryo are able to differentiate to generate primitive ectoderm, which ultimately differentiates into three primary germ layers to lead the formation of gastrula^15^. Since Trim59 deficiency affects the mouse development at the gastrulation, it is possible that Trim59 deficiency may affect the differentiation of ICM. To test this, we examined in vitro outgrowth of embryos at E3.5. At this stage, morula develops into blastocyst, and EPI is formed^16^. Female *Trim59*+*/−* mice were first superovulated, and then embryos were dissected from the uterus in PBS buffer. A total of 17 outgrowths were generated. Each outgrowth was divided into two parts, one for genotyping, other blastocysts to establish stem cell lines. PCR revealed that 12 of 17 outgrowths were *Trim59*+/+or *Trim59*+*/−* genotype, and other were *Trim59−/−* genotype (Fig. 4a, b). Importantly, while all *Trim59*+/+ 12 outgrowths grew to form stable stem cell lines, all of five *Trim59−/−* outgrowths failed to do this (Fig. 4a, b), indicating that Trim59 is necessary for the growth and differentiation of ICM.

The expression of transcription factor Oct4 (octamer-binding transcription factor 4) is essential for the differentiation of blastocysts^17^. Altered expression of Oct4 also marks the abnormal differentiation of blastocysts. Thus, we assessed Oct4 in mouse early embryos. Consistent with other data, Oct4 expression was restricted to ICM and EPI in wt blastocysts (Fig. 4c). Notably, during this stage, Trim59 could co-express with Oct4 in the ICM and EPI (Fig. 4c). However, the expression region of Oct4 remarkably decreased during gastrulation in *Trim59−/−* as compared to *Trim59*+/+ embryos (Fig. 4d), further indicating that ICM differentiation is abnormal in *Trim59−/−* blastocyst stage. Taken together, our data suggest that Trim59 is a critical factor for ICM differentiation in blastocyst stage embryos.

### Trim59 deficiency disturbs F-actin polymerization during ICM differentiation

**Fig. 5:**
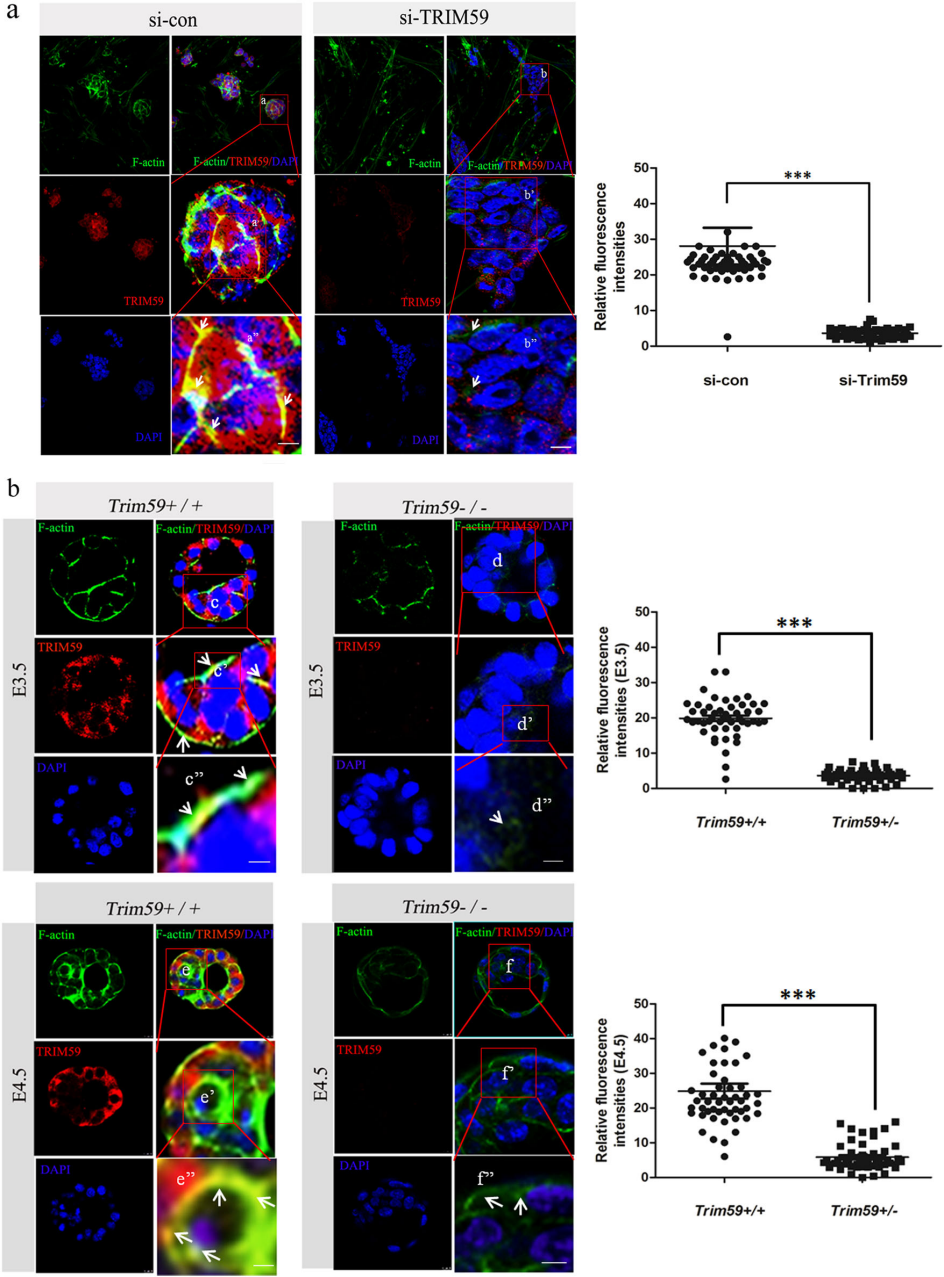
Trim59 deficiency affects F-actin polymerization. **a** Immunofluorescence assay of Trim59 and F-actin in F1 ESCs transfected with Trim59 siRNA (si-TRIM59) or siRNA control (si-con). **b** Confocal fluorescence images of embryos at E3.5 and E4.5 stained with Alexa488. Images were acquired using a Bio-Rad Radiance 2100 confocal microscope with a Zeiss 63× oil immersion objective. Scale bar, 10 µm; arrows indicate F-actin polymerization. *P < 0.05, **P < 0.01, ***P < 0.005; Ns no significance

**Fig. 6:**
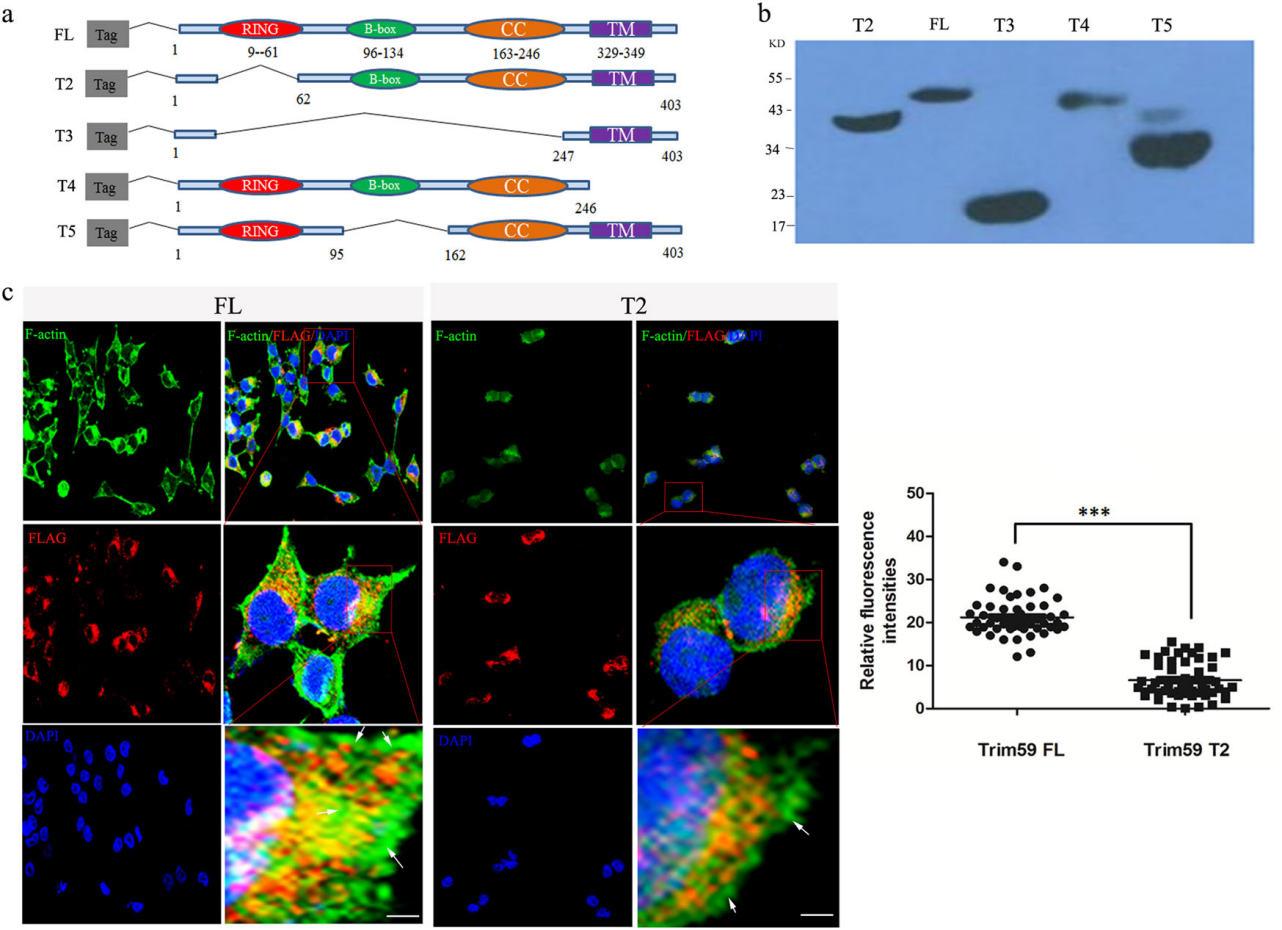
Trim59 RING-finger domain is required for F-actin assembly. **a** Schematic representation of full-length Trim59 and its domain deletion mutants. RING RING-finger domain, B-box B-box domain, CC coiled-coil domain, TM transmembrane domain. FL full-length Trim59, T2 RING-finger domain deleted fragment, T3 RING-finger domain, B-Box domain and coiled-coil domains deleted fragment, T4 C terminal TM deleted fragment, T5 B-box deleted fragment. **b** Immunoblotting of Trim59FL and its fragments. The expression of Trim59 FL and its fragments was analyzed by anti-FLAG antibody. **c** Immunofluorescence assay of F-actin in FLAG-tagged Trim59FL or its fragments transfected HEK293T cells. Scale bar, 10 µm. *P < 0.05, **P < 0.01, ***P < 0.005; Ns no significance

Since Trim59 plays a critical role in the blastocyst development stage, next question asked is how Trim59 regulates the formation of blastocysts. We first employed a yeast two-hybrid method to find target molecules of Trim59. Trim59 potentially interacted with at least 20 proteins (only eight proteins were listed) (Supplementary Table S1), some of which are relative to the function of cytoskeleton in early embryos, including actinin alpha 1(ACTN1), phospholipid scramblase 1 (PLSCR1), and F-actin binding protein (TRIOBP). Immunoprecipitation-mass spectrometry further revealed that Trim59 interacted with myosin and F-actin capping protein (Supplementary Figure S3), which were also associated with the cellular cytoskeleton functions during early embryo development. Deficiency or dysregulation of the genes involved in the regulation of actin skeleton may lead to impaired embryonic development and lethality^18^. So we proposed that the effect(s) of Trim59 on blastocyst development may be through modulating actin cytoskeleton. To test this hypothesis, we employed a loss-of-function experiment. In untreated F1 ESCs, F-actin was polymerized at the cell–cell junction and at the edge of the cultivated limbal stem cell (Fig. 5a). However, while F1 ESCs were treated by silencing Trim59, Trim59 expression was downregulated (Supplementary Figure S4). F-actin polymerization at the cell–cell junction and at the edge of the cultivated limbal stem cell was interrupted (Fig. 5a). We then examined blastocysts of *Trim59−/−* embryos. At E3.5 and E4.5, the blastocysts in *Trim59−/−* embryos weakened the assembly of F-actin at the edge of cell–cell junction as compared to wt embryos (Fig. 5b). Trim59 contains three functional domains including a RING-finger, a coiled-coil region, and a B-box domain. We next further determined which domain of Trim59 induced the polymerization of F-actin. FLAG-tagged full-length (FL) Trim59 and four different deletion mutants of Trim59 were generated (Fig. 6a, b). Only Trim59 FL and Trim59 fragments (T4 and T5) that contain a RING-finger domain could promote the assembly of F-actin at the boundary of cells and maintain cells in the stretched state, whereas in Trim59 fragments T2 and T3 transfected HEK293T cells, which were absence of RING-finger domain, there was a decreased immunofluorescence intensity, rounded cells edge, degraded diopter, and concomitant morphological changes as compared to cells transfected by fragments containing RING-finger domain (Fig. 6c; Supplementary Figure S5). Thus, RING-finger domain of Trim59 is necessary for the regulation of F-actin assembly. Taken together, the effects of Trim59 on ICM differentiation are through disturbing F-actin polymerization.

### Trim59 promotes polymerization of F-actin via WASH K63-linked polyubiquitination

**Fig. 7:**
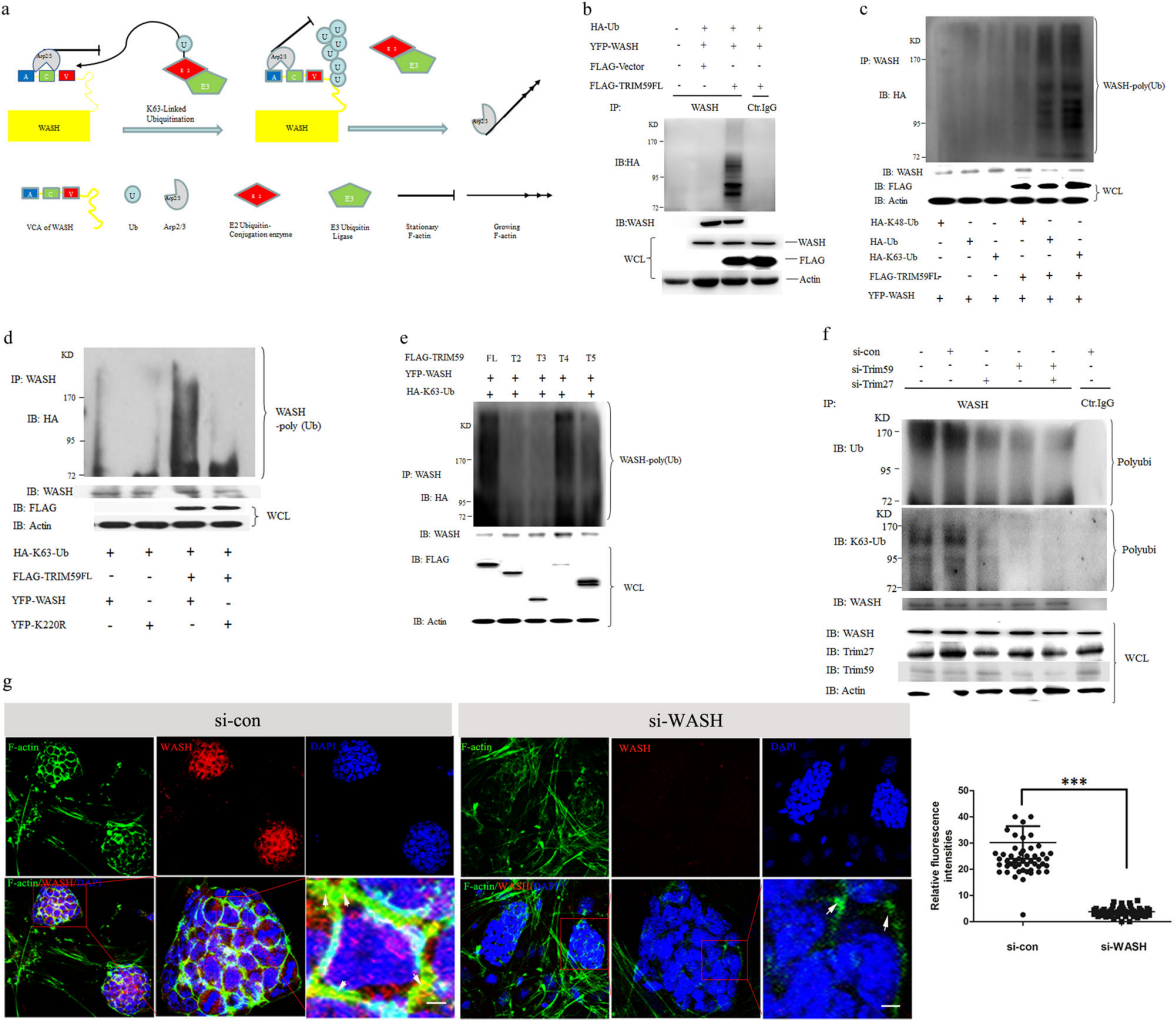
Trim59 promotes WASH K63-linked ubiquitination. **a** Model of WASH, Arp2/3, E2, E3, and K63 ubiquitin regulation on F-actin polymerization. **b** Immunoblotting of WASH total Ubiquitin in cotransfected HEK293T cells. HEK293T cells were cotransfected with YFP-WASH and with (+) or without (*−*) FLAG-Trim59FL or FLAG-Vector as well as with HA-Ub. Immunoprecipitation was performed by anti-WASH. Trim59 and WASH were detected by anti-FLAG and anti-WASH antibodies. The polyubiquitination of WASH was detected by anti-HA. **c** Immunoblotting of WASH K63-linked ubiquitin in cotransfected HEK293T cells. HEK293T cells were cotransfected with YFP-WASH and with (+) or without (*−*) FLAG-Trim59 as well as with HA-Ub, HA-K48-Ub, or HA-K63-Ub. Immunoprecipitation was performed by anti-WASH. Trim59 and WASH were detected by anti-FLAG and anti-WASH antibodies. The polyubiquitination of WASH was detected by anti-HA. **d** Immunoblotting of WASH K63-linked ubiquitin in cotransfected HEK293T cells. HEK293T cells were cotransfected with YFP-WASH or YFP-K220R and with (+) or without (−) FLAG-Trim59 as well as with HA-K63-Ub. Immunoprecipitation was performed by anti-WASH. Trim59, WASH and WASH variant were detected by anti-FLAG and anti-WASH antibodies. Polyubiquitination of WASH was detected by anti-HA. **e** Immunoblotting of WASH K63-linked ubiquitin in cotransfected HEK293T cells. HEK293T cells were cotransfected with FLAG-Trim59FL and its fragments (T2 toT5) and with YFP-WASH (+) as well as HA-K63-Ub (+). Immunoprecipitation was performed by anti-WASH. Trim59, Trim59 fragments and WASH were detected by anti-FLAG and anti-WASH antibodies. Polyubiquitination of WASH was detected by anti-HA. **f** Immunoblotting of total Ub and K63-Ub of endogenous WASH from F1 ESCs after treated with specific Trim59 and Trim27 siRNA.Immunoprecipitation was performed by anti-WASH. Trim59, Trim27, and WASH were detected by anti-Trim59, anti-Trim27, and anti-WASH antibodies. Immunoblot analysis of Ub-WASH (top blot), K63-Ub-WASH (below blot) by anti-Ub and anti- K63 antibodies. **g** Immunofluorescence assay of F-actin assembly in WASH siRNA or siRNA control transfected F1 ESCs. Scale bar, 10 µm. *P < 0.05, **P < 0.01, ***P < 0.005; Ns no significance. Polyubi. polyubiquitination, IP immunoprecipitation, IB immunoblot assay, Actin a loading control, WCL whole-cell lysates, Ctr.IgG isotype control IgG

Previous studies have shown that TRIM proteins with RING-finger domain may act as an E3 ubiquitin ligase^19^. Next, we investigated whether Trim59 mediated F-actin polymerization is through its ubiquitin ligase activity. We first examined which protein was involved in Trim59 mediated F-actin polymerization. WASH, a member of Wiskott–Aldrich syndrome protein (WASP) family, plays an important role in regulating the polymerization of F-actin (Fig. 7a)^20^. Thus we hypothesized that Trim59 could promote WASH ubiqutination through ubiqutin-protease system. Indeed, Trim59 promoted WASH protein polyubiquitin (Fig. 7b). Because ubiquitin linkages via lysine 48 (K48) or 63(K63) can differentially address the proteins for 26S proteasomal degradation or a common activating signal^21^, we transfected HEK293T cells to express WASH and Trim59 in the presence of vectors encoding K48-linked ubiquitin or K63-linked ubiquitin. Trim59 resulted in more K63-linked ubiquitin but not K48-linked ubiquitin on WASH protein (Fig. 7c). Meanwhile we also utilized a K220R WASH mutant as a control, which is an ubiquitin variant and unable to produce a specific ubiquitin chain^22^. Trim59 did not increase WASH K220R mutant K63-linked ubiquitin (Fig. 7d). RING-finger domain deletion mutants T2 and T3 failed to promote WASH K63-linked ubiquitin (Fig. 7e). We also immunoprecipitated endogenous WASH and measured total Ub, K63-linked or K48-linked ubiquitin associated with WASH by silencing the expression of Trim59 with specific siRNA. The total Ub and K63-linked ubiquitin on the WASH protein were obviously decreased after silencing Trim59 (Fig. 7f). No K48-linked ubiquitin was found in both control and Trim59 siRNA transfection groups (Supplementary Figure S6). Since WASH was previously reported to be ubiquitinated on Lys 220 by Trim 27^22^, we also compared the effect(s) of Trim59 and Trim27 on WASH. Trim59 had stronger effects on the WASH ubiquitination than Trim27 (Fig. 7f). Taken together, these data suggest that WASH may be involved in Trim59-mediated assembly of F-actin. Indeed, when the expression of WASH protein was interrupted by specific siRNA in vitro, WASH was obviously downregulated (Supplementary Figure S7). Meanwhile the stem cell mass was irregular than those by control transfection (Fig. 7g). Especially some smaller cell masses appeared after silencing WASH (Fig. 7g). Meanwhile, F-actin polymerization decreased at the cytomembrane and cell–cell junction (Fig. 7g). These results suggest that Trim59*-*mediated assembly of F-actin is dependent on WASH K63-linked ubiquitination.

## Discussion

In this study, we found that Trim59 is a critical regulator for early embryo development from blastocyst stage to gastrula through modulating F-actin assembly. *Trim59−/−* embryos are failure for normal embryogenesis and have a reduced expression of primary germ layer formation-associated genes including *Brachyury*, *lefty2*, *Cer1*, *Otx2*, *Wnt3*, and *BMP4*. Trim59 deficiency may disturb F-actin polymerization during ICM differentiation. We also demonstrate that the effect of Trim59 on F-actin polymerization is through WASH K63-linked ubiquitination. Overall, these results provide a molecular basis for early embryonic development. More broadly, our findings are relevant to understand the impact of Trim59 on human infertility and embryonic lethality.

TRIM family members have been implicated in a variety of biological processes, such as the regulation of differentiation and development^23^. Several members of TRIM proteins have been found to play a crucial role during early embryonic development. Trim33 regulates ectodermal induction by functioning as a smad4 ubiquitin ligase^24^. Trim71 has RING-dependent ubiquitin ligase activity. *Trim71−/−* embryos present a highly penetrant closure defect of the cranial neural tube, and cease development and die between E9.5 and E11.5^25^. TRIM36 also markedly and specifically inhibits somite formation and vegetal microtubule polymerization^26^. Trim36-depleted embryos are disrupted in the development of cortical rotation in a manner dependent on ubiquitin ligase activity. We here found that Trim59 plays a critical role in early embryos development from blastocyst stage into gastrula. Trim59 knockout affects primary germ layers formation at the beginning of gastrulation. Thus, multiple members of TRIM family may be involved in early embryos development.

Our studies suggest that Trim59 is necessary for the formation of primary germ layers, which happens at the beginning of gastrulation. Transition from blastocyst to gastrula is a remarkably elaborate process involving a multiple of genes such as *Brachyury*, *Otx2*, *Cer1*, and *Lefty2*^27^. *Brachyury* is a key player in mesoderm formation. *Brachyury* deficiency fails to elongate along the anterior–posterior axis and their embryos cannot develop mesodermal extraembryonic tissues. Our data show that *Brachyury* is not detected in *Trim59−/−* embryonic blastopore, suggesting that Trim59 deficiency affects the mesoderm formation. *Otx2*, *Cer1*, and *Lefty2* belong to AVE genes, expression of these genes may not also be detected in Trim59-deficient embryos, indicating that AVE formation may be regulated by Trim59. Many signaling molecules participate in regulating early embryos development such as BMP, Nodal, Wnt, and FGF. Wnt3 activity derived from the posterior visceral endoderm has a temporal role in establishing the primitive streak^28^. Wnt3 co-receptor Lrp6 knockout embryos fail to establish a primitive streak^29^. Extra-embryonic ectoderm (ExE) expresses BMP4, which is involved in many steps in pregastrulation development. The loss of BMP4 functions leads to gastrulation defects^30^. In *Trim59−/−* embryos, there have been a remarkably reduced expression in both Wnt3 and BMP4. The expression region of BMP4 also moves to the EPI. Oct4 is expressed in mouse totipotent embryonic stem and germ cells, and totipotent cells differentiate into somatic lineages occurred at the blastocyst stage and during gastrulation. When Oct4 is deleted, embryonic stem (ES) cells lose the capacity to self-renew and subsequently differentiate into extra-embryonic trophectoderm. *Oct4−/−* embryos die at peri-implantation stages due to the conversion of ICM into trophectoderm^31^. At E6.5 and E7.5, Trim59 knockout also decreases the expression of Oct4.

Our mechanistic studies uncovered that K63-linked ubiquitination of WASH by Trim59 is required for polymerization of F-actin during gastrulation. Actin microfilaments are the major regulators and play a crucial role in cell morphology and mobility. We found that Trim59, as an E3 ligase family member, promotes WASH K63-linked polyubiquitination through RING finger domain. Recent studies have also indicated that regulation of WASH-dependent actin polymerization is based on K63 ubiquitination in WASH^22^. WASH is a member of the Wiskott–Aldrich syndrome protein family consisting of WASP/N-WASP, WAVE, WHAMM, JMY, and WASH^32^. It contains a carboxyterminal VCA (verprolin homologous or WH2, central hydrophobic, and acidic) motif that binds to actin and Arp2/3 complex to stimulate actin filament nucleation. Ubiquitination is a posttranslational modification that can have pleiotropic effects on its substrates depending on the length and type of ubiquitin chains. Studies have shown that K63-linked ubiquitination typically acts as a signaling event to modify function, such as altering protein–protein interactions, protein conformations, or targeting proteins for lysosomal delivery. Our data demonstrate that WASH K63-linked ubiquitination by Trim59 determines the polymerization of F-actin during gastrulation.

## Materials and methods

### Generation of trim59 knockout mice

In brief, Trim59 gene was retrieved from a 129/sv BAC clone bMQ452g13 (provided by Sanger Institute) by a retrieval vector containing two homologous arms. After correct recombination, this vector contains 11.3 kb of genomic sequence including part of intron II, exon III and 3.5 kb downstream sequences. 147 bp intron II and the entire coding region of Trim59 were then deleted and replaced with a loxP-Neo-loxP cassette. The targeting construct that contains a neo cassette for positive selection and a herpes simplex virus-thymidine kinase expression cassette for negative selection was linearized with *Not* I and electroporated into C57BL/6-derived B6/BLU embryonic stem (ES) cells. 96 ES cell clones were selected and verified for correct recombination with long range PCR and Southern blot analysis. Correctly targeted ES cells were injected into C57BL/6J blastocysts followed by transfer to pseudopregnant mice. Chimerical male mice identified by PCR were bred to C57BL/6J females to generate F1 offspring. Germ line transmission of the targeted Trim59 allele was verified by PCR analysis of tail DNA from F1 offspring with agouti coat color. All procedures were conducted in accordance with Institutional Animal Care and Use Committee of Model Animal Research Center. Primers used in this study were listed in supplementary Table S2a.

### Reagents and plasmid constructs

Mouse Trim59, Trim27, WASH, and control siRNAs were purchased from Ribo, Shenzheng, China. Anti- Trim59 (ab69639, Abcam), anti-Trim27 (A6405, Abclonal), anti-WASH (SAB-4200552, Sigma), anti-Oct4 (ab18976, Abcam), anti-FLAG (M20008, Abmart), anti-HA (#T501-1, Signalway Antibody), anti-Ub (YT4793, Immunoway), anti-K48 (#4289, Cell Signaling Technology), anti-K63 (BML-PW0600, Enzo), anti-ACTR2 (ab134082, Abcam), anti-MYH9 (ab138498, Abcam), anti-CAPZA1 (ab166892, Abcam), anti-CAPZB (ab175212, Abcam), anti-β-actin (SC-81178, Santa), Phalloidin-iFluor 488 (ab176753, Abcam), DAPI (#4083, Cell Signaling Technology), Alexa Fluor 488 Conjugate (#4416, #4408, Cell Signaling Technology), and Alexa Fluor 594 Conjugate (#8890, #8889, Cell Signaling Technology) antibodies were purchased.

Murine FL Trim59 clone was obtained from the ATCC. Trim59 mutants were constructed by performing PCR with four primers according to a previous method^33^. All primers used in this study are listed in supplementary Table S2b. YFP–WASH and YFP-WASH K220R mutant were from Patrick Ryan Potts, UT Southwestern Dallas, TX 75390, USA; plasmids encoding hemagglutinin (HA)-tagged ubiquitin (HA-Ub), HA-K48-Ub, and HA-K63-Ub were obtained from Y. Xiong (University of North Carolina, Chapel Hill).

### Embryos dissection and genotyping

The female *Trim59*+*/*- mice were first superovulated by intraperitoneal injection of 5 IU pregnant mare serum gonadotropin (PMSG), and followed by injection of 5 IU human chorionic gonadotropin (hCG) 48 h later, and then mated with male *Trim59*+*/−* mice. Females were screened for vaginal plugs following morning (E0.5). Embryos were collected from the uterus and part of embryo tissue was used for genotyping.

### RT-PCR and qRT-PCR

Briefly, total RNA was extracted from the cells, tissues and organs using TRIzol reagent (Invitrogen Corp). First-strand cDNA was generated from the total RNA using oligo-dT primers and reverse transcriptase (Invitrogen Corp). The PCR products were visualized on 1.0% (wt/vol) agarose gel. Real-time PCR was conducted using QuantiTect SYBR Green PCR Master Mix (Qiagen) and specific primers in an ABI Prism 7000 analyzer (Applied Biosystems). The fold changes were calculated using the ∆∆C_t_ method according to the manufacturer’s instructions (Applied Biosystems). All the reactions were run in triplicate. Primer sequences are listed in Supplementary Tables S2c and d.

### RNA interference

F1 ESCs were transfected with murine Trim59, Trim27, WASH, or control siRNAs by Hiperfect Transfection Reagent (siRNA transfection) (Qiagen, Valencia, CA, USA). siRNA sequences are listed in Supplementary Table S2e.

### Immunoblot, immunoprecipitation, and liquid chromatography-tandem mass spectrometry

For immunoprecipitation, HEK293T cells were transfected with YFP -WASH, YFP-WASH K220R mutant, FLAG-Trim59, FLAG-vector, HA-Ub, HA-K48 Ub, and/or HA-K63 Ub vectors for 24 h. The cells were lysed with cell lysis buffer (Cell Signaling Technology). Immunoprecipitation (IP) was performed as described by the manufacturer (Thermo Scientific, USA).

For liquid chromatography-tandem mass spectrometry (LC-MS/MS) analyses, HEK293T cells were transfected with FLAG-Vector or FLAG-Trim59 Vector for 24 h. FLAG-tagged primary antibody was used for immunoprecipitation. Trim59-associated complexes were eluted with SDS sample buffer, separated by SDS-PAGE, and stained with Fast Silver Stain Kit (Beyotime Biotechnology).The gel lanes were excised for LC-MS/MS analyses by Peking University Health Science Center (China) using a nanoflow-HPLC system interfaced to electrospray Q-TOF tandem mass spectrometers. Protein identification was achieved via peptide MS/MS spectra by using the Mascot software for searching the NCBI non-redundant protein database.

For immunoblot, hybridizations with primary antibodies were conducted for overnight at 4 °C. The protein–antibody complexes were detected using peroxidase-conjugated secondary antibodies (Boehringer Mannheim) and enhanced chemiluminescence (Amersham).

### Histology, immunofluorescence, and image analyses

For H&E staining, embryos were fixed in 10% formalin-buffered saline and embedded in paraffin, 5 µm sections were cut and stained.

For immunofluorescence staining, embryos collected from uterus were fixed for 30 min in 4% PFA and were permeabilized in 0.1% Triton X-100 for 30 min. 5% goat serum was used for blocking. Primary anti-Trim59 (1:500) was added for 2 h. Then coverslips were stained with phalloidin (1:200) and Alexa 488-conjugated secondary antibodies for 1 h. Nucleus were stained with DAPI for 3 min. Embryos were placed in glycerin droplet for fluorescence microscopy immediately.

For immunofluorescence staining of cells, F1 ESCs were cultured on slides and transfected by siRNA for 72 h. ES cells were fixed for 20 min in PBS containing 4% PFA, and then were permeabilized in 0.1% Triton X-100 for 30 min. 5% goat serum was used for blocking. Primary anti-Trim59 (1:500) or anti-WASH (1:500) antibodies were added for 2 h. Then the coverslips were stained with phalloidin (1:200) and Alexa 488-conjugated secondary antibodies for 1 h. Immunofluorescence images were obtained with laser scanning confocal microscopy. For image analyses, F-actin polymerization was quantified by ImageJ software. Mean signal intensities in the plotted areas were calculated by the ImageJ software. More than ten views were used for the analysis each image, and five images of different treatment groups were selected randomly to perform analysis.

### Mouse embryo stem cell culture

Intact blastocysts from *Trim59*+*/−* crosses were seeded on the feeder layers of mitomycin C-treated MEF in the ES medium consisting of knockout DMEM (Gibco), 20% KSR, supplemented with 1000 units/ml mouse leukemia inhibitory factor (LIF) (ESGRO, Chemicon), 0.1 mM non-essential amino acid (NEAA), 1 mM l-glutamine, 0.1 mM β-mercaptoethanol, penicillin (100 μg/ml) and streptomycin (100 μg/ml). Half of the medium was changed daily. Approximately 10 days after seeding, inner cell mass outgrowth was mechanically removed and divided into two parts: one part was collected for genotyping and the other was digested with 0.25% trypsin–EDTA into small clumps for ES cell line derivation. Digestion was halted with trypsin inhibitor and the outgrowths reseeded on fresh feeder cells. All ES cell lines then were massaged and cultured in 20% FBS (Hyclone) ES medium (instead of KSR ES medium). ES cell lines were stored in freezing medium (ES medium supplemented with 10% DMSO and 40% FBS), and frozen in liquid nitrogen.

### In situ hybridization

Embryos were harvested through dissected from uterus then dissected into PBS, amniotic and yolk sac were fixed in 4% PFA for 3 h, and dehydrated in ethanol at an increasing series of concentrations from 70 to 100%. Then embryos were rinsed in methylsalicylate and embedded in paraffin. The samples were sectioned into 5 μm slides and then hybridized with corresponding probes. Digoxigenin-labeled anti-sense RNA probes to mouse *Oct4*, *Brachyury*, *Bmp4*, *Wnt3*, *Cer1*, *Lefty2*, *Otx2*, and *Trim59* gene were used in situ hybridization. The sense probes were used as negative controls. The sequence of associated gene probes were listed in Supplementary Table S2f.

### Yeast two-hybrid analyses

Yeast two-hybrid analysis was performed by Beijing Proteome Research Center. The ProQuest yeast two-hybrid system (Y2H) (Invitrogen) was employed to assess interactions between Trim59 and other subfamily members. Trim59 was first cloned into pDBleu as a bait plasmid pDBleu-Trim59. A mouse embryos cDNA library fused to GAL4AD of pEXP-AD502 (Invitrogen) was screened for proteins that interact with Trim59 using the ProQuest Two-Hybrid System (Invitrogen). The detailed method of yeast two-hybrid screening has been described previously (Invitrogen). Briefly, the pEXP-AD502 plasmid and the pDELeu-Trim59 bait plasmid were co-transformed into AH109 yeast cells. Transformed yeast cells were plated on medium lacking histidine or uracil or medium containing 5-fluoroorotic acid (5FOA). The transformed yeast cells were also plated on YPAD plates to further conduct β-galactosidase assays. A total of 1 × 10e7 library clones were screened for growth on selective media and assayed for β-galactosidase activity. pEXP-AD502 cDNA plasmids were recovered by bacterial transformation of DNA isolated from positive yeast colonies. The candidate pEXP-AD502 cDNA plasmids were retransformed into yeast cells with the empty pDEST32 vector or pDELeu-Trim59 plasmid encoding irrelevant bait to exclude false-positives. Inserts of true-positive pEXP-AD502 cDNA clones were characterized by sequence analysis.

### Statistical analyses

All quantitative data were expressed as mean ± SEM. Significance was evaluated with a two-tailed unpaired Student’s *t*-test or the Mann–Whitney *U*-test. Excel and Prism Version 5 software (GraphPad) were used for statistical evaluation. A 95% confidence interval was considered significant and was defined as *P* < 0.05.

## Electronic supplementary material

supplemental data
