# Supplementary material for: Embryonic lethality in mice lacking Trim59 due to impaired gastrulation development
Source: Cell Death Dis. 2018 Feb 21;9(3):302. doi: 10.1038/s41419-018-0370-y (PMC5833458; doi:10.1038/s41419-018-0370-y)
Supplement: Supplementary file 1 — supplemental data [file 41419_2018_370_MOESM1_ESM.pdf]

Supplementary Data for

Embryonic Lethality in Mice Lacking Trim59 Due to Impaired Gastrulation Development

Xiaomin Su[1,2,3#], Chenglei Wu[1,2#], Xiaoying Ye[4#], Ming Zeng[4], Zhujun Zhang[1], Yongzhe Che[1], Yuan Zhang[1], Lin Liu[3,4], Yushuang Lin[5]*, Rongcun Yang[1,2,3]*

[1] Department of Immunology, Nankai University School of Medicine, Nankai University, Tianjin, China.

[2] Key Laboratory of Bioactive Materials Ministry of Education, Nankai University, Tianjin, China.

[3] State Key Laboratory of Medicinal Chemical Biology, Nankai University, Tianjin, China.

[4] College of Life Sciences, Nankai University, Tianjin, China.

[5] Shandong Provincial Key Laboratory of Animal Cells and Developmental Biology.

[#]These authors contributed equally to this work.

*Correspondence and requests for materials should be addressed to Rongcun Yang or Yushuang Lin. (email: ryang@nankai.edu.cn or linyushuang@sdu.edu.cn).

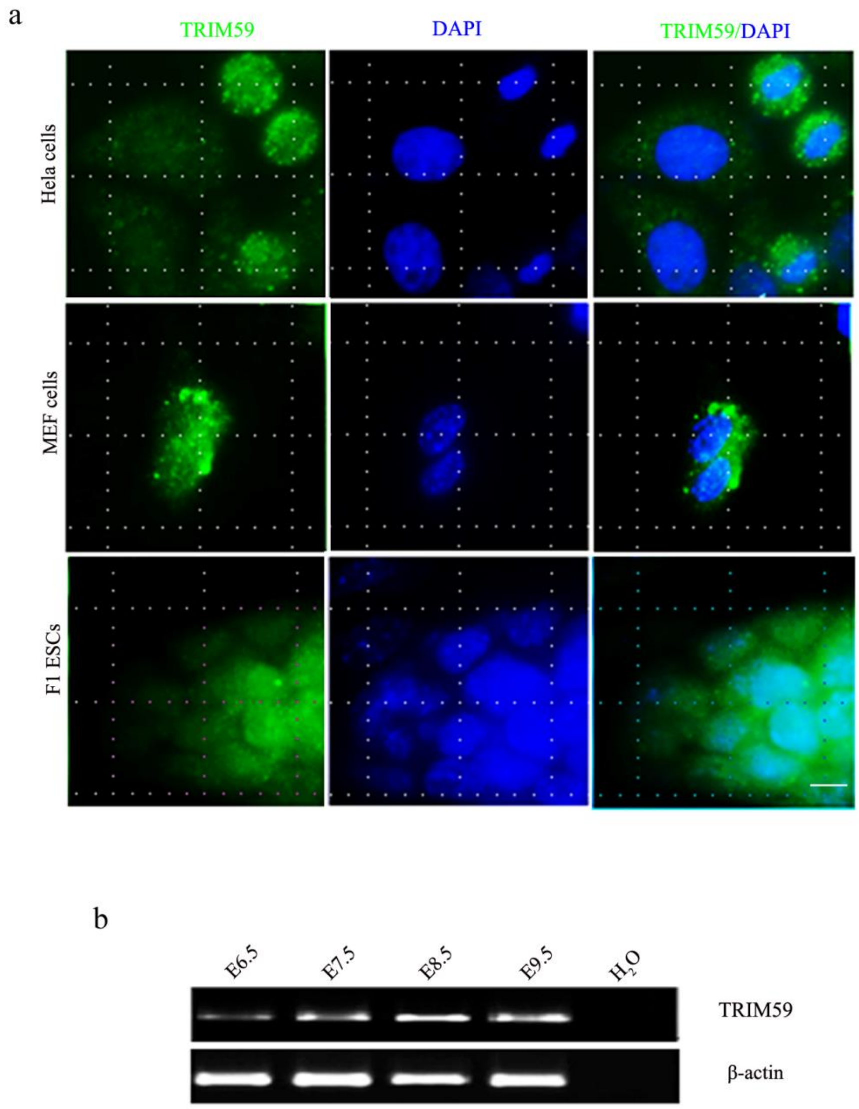

Supplementary Figure S1 Expression of Trim59 in different cell lines and mouse early embryos. a Immunofluorescence assay of Trim59 in Hela cells (human cervical cancer cell), MEF (murine Embryonic Fibroblast) cells and F1 ESCs. Trim59 (green) and chromatin (blue). Images were acquired using a Bio-Rad Radiance 2100 confocal microscope with a Zeiss 63× oil immersion objective. Scale bar, 10 μm. **b** RT-PCR of Trim59 in mouse early embryos from E6.5 to E9.5. β-actin, a loading control.

a

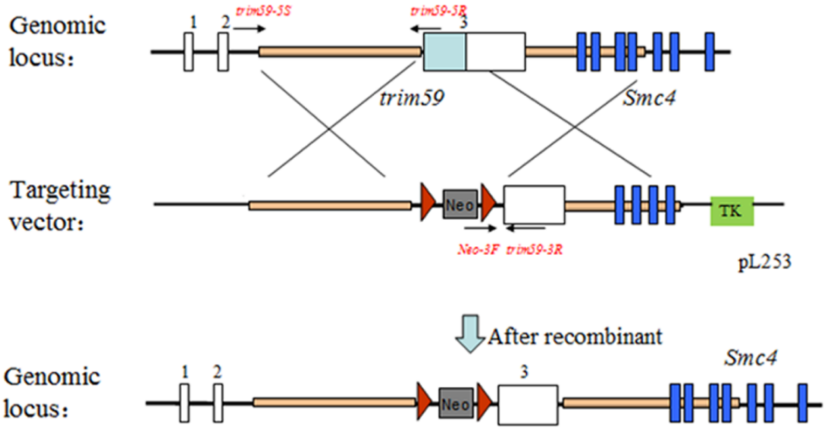

b

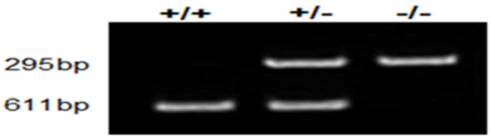

Supplementary Figure S2 Generation and identification of *Trim59* knockout mice. a Strategy for generating *Trim59* KO mice. *Trim59* KO mice were generated according to the described protocol in materials and methods. **b** PCR of mouse tail genome DNA. Total tail genome DNA was extracted using a DNA kit and PCR was performed. +/+, wild-type mouse; +/–, heterozygote mouse; –/–, *Trim59* KO mouse.

a

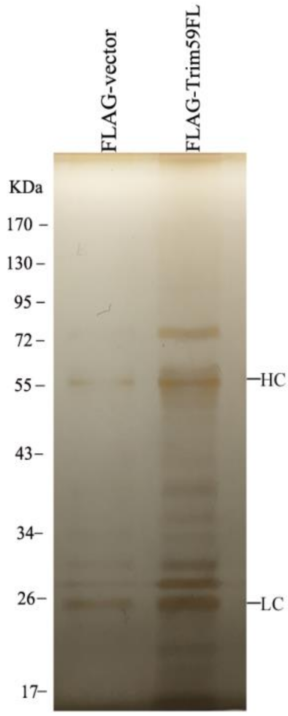

c

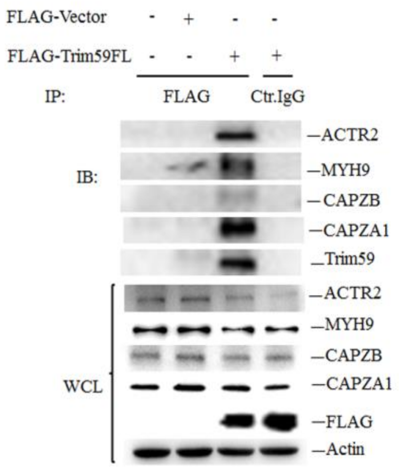

b

Name	Accession number	Description
**CAPZB**	**NM_001206540.2**	***Homo sapiens* capping actin protein of muscle Z-line beta subunit**
**CAPZA1**	**NM_006315.2**	***Homo sapiens* capping actin protein of muscle Z-line alpha subunit 1**
**ACTR2**	**NM_001005386.2**	***Homo sapiens* ARP2 actin related protein 2 homolog**
**MYL6**	**NM_021019.4**	***Homo sapiens* myosin light chain 6**
**MYH9**	**NM_002473.5**	***Homo sapiens* myosin heavy chain 9**
**MYH10**	**NM_001256012.1**	***Homo sapiens* myosin heavy chain 10**

Supplementary Figure S3 Interaction of Trim59 and actin-associated proteins. **a** Immunoprecipitation analyses. HEK293T cells were transfected with FLAG-vector or FLAG-Trim59. After immunoprecipitation by anti-FLAG, proteins were separated by SDS-PAGE, and then stained using silver staining kit. Asterisks indicate proteins which only appeared in Trim59 overexpressing group. HC, antibody heavy chain; LC, antibody light chain. **b** Proteins which are relative to the functions of cytoskeleton which only appeared in FLAG-Trim59 overexpressing group. The gel lanes containing the immunopurified samples were excised for Liquid Chromatography-

tandem Mass (LC-MS/MS) analysis. **c** Immunoprecipitation analyses. HEK293T cells were transfected with FLAG-vector (+) or FLAG-Trim59FL (+). After immunoprecipitation by anti-FLAG, Trim59 was detected by anti-Trim59 antibody. Trim59 binding protein ACTR2, MYH9, CAPZA1 and CAPZB were detected by anti- ACTR2, anti-MYH9, anti-CAPZA1 and anti-CAPZB. IP, immunoprecipitation; IB, immunoblot assay; WCL, whole cell lysate; Actin, a loading control; Ctr.IgG, isotype contro IgG.

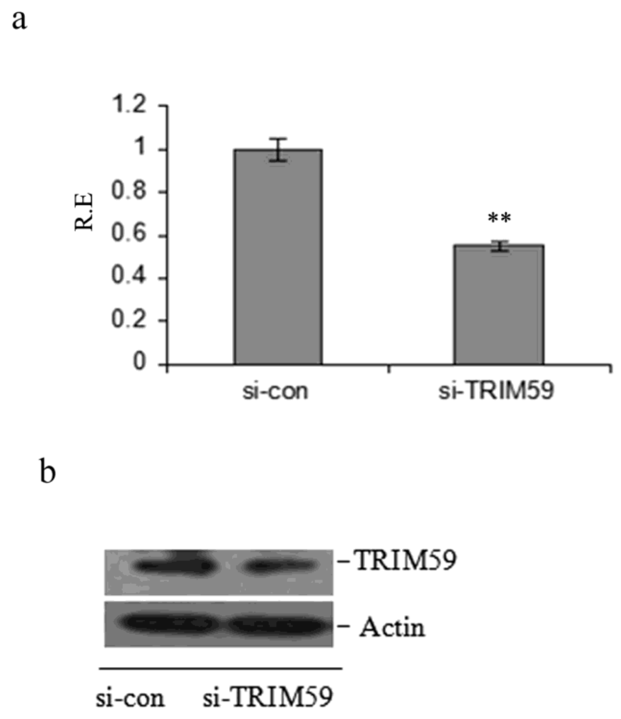

Supplementary Figure S4 Expression of Trim59 in siRNA silencing mouse embryonic stem cells. a qRT-PCR of Trim59 in the F1 ESCs with (si-TRIM59) or without (si-Con) mouse Trim59 siRNA treatment. *p<0.05 and **p<0.01(t-test). **b** Immunoblot of Trim59 in the F1 ESCs with (si-TRIM59) or without (si-Con) mouse Trim59 siRNA treatment. Actin, a loading control.

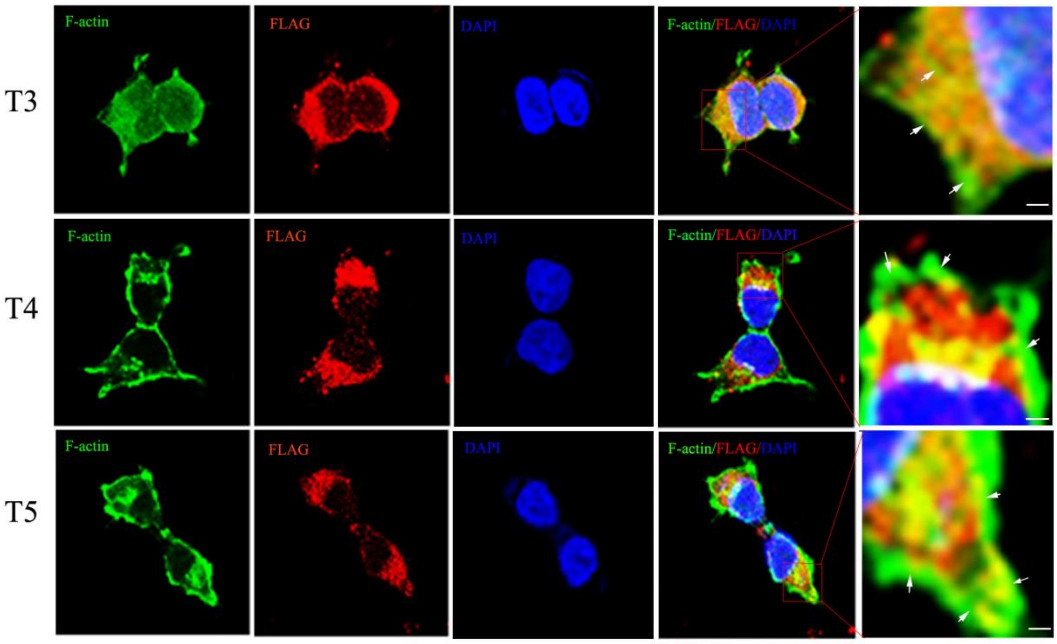

Supplementary Figure S5 Trim59 RING-finger domain is required for F-actin assembly. HEK293T cells were transfected with expression vectors encoding FLAG-tagged Trim59 domain mutants for 24hrs. The expression of FLAG-tag Trim59 domain deleted mutants (T3, T4, T5) and the F-actin assembly were analyzed by immunofluorescence. F-actin (green), Trim59 mutants (red) and chromatin (blue). Images were acquired using a Bio-Rad Radiance 2100 confocal microscope with a Zeiss 63 × oil immersion objective; scale bar, 10 μm; zoom: ×4.

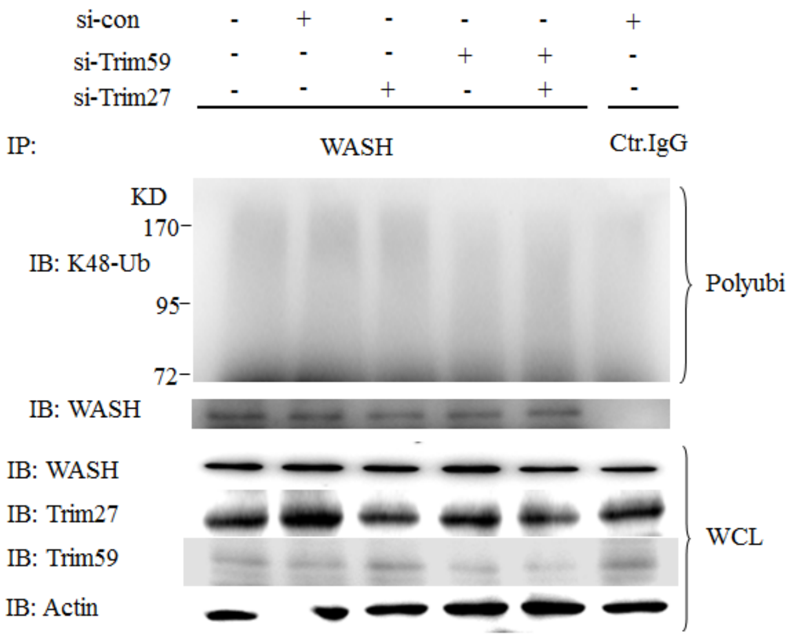

Supplementary Figure S6 Immunoblot of K48-linked ubiquitination of endogenous WASH. Immunoblot analysis of K48-linked ubiquitination (K48-Ub) of endogenouse WASH which was immunoprecipitated in F1 ESCs after treated with mouse Trim59 siRNA (top). Trim59, WASH and actin (below) were detected in the same cells without immunoprecipitation. Polyubi., polyubiquitination; IP, immunoprecipitation; IB, immunoblot assay; Actin, a loading control; WCL, whole cell lysates; Ctr.IgG, isotype control IgG.

a

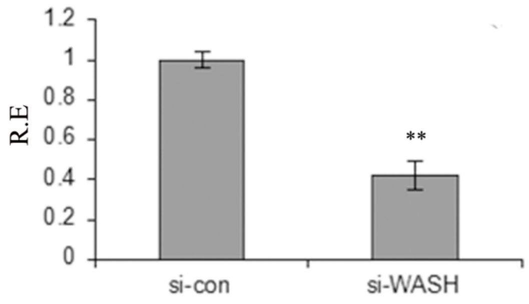

b

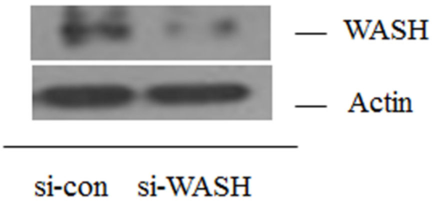

Supplementary Figure S7 Expression of WASH after siRNA treatment. a qRT-PCR of WASH in F1 ESCs with (si-WASH) or without (si-Con) mouse WASH siRNA.*p<0.05 and **p<0.01(t-test). **b** Immunoblot of WASH in F1 ESCs with (si-WASH) or without (si-Con) mouse WASH siRNA. Actin, a loading control.

Supplementary Table S1

Table S1 Proteins that potentially interact with Trim59 (data from yeast two-hybrid)

Name	Accession number	Description
ACTN1	NM_001102	*Mus musculus* actinin, alpha 1
ARNT	NM_178427	*Mus musculus* aryl hydrocarbon receptor nuclear translocator
CD74	NM_004355	*Mus musculus* CD74 antigen
HOMER3	NM_004838	*Mus musculus* homer homolog 3
PLSCR1	NM_021105	*Mus musculus* phospholipid scramblase 1
RPS2	NM_002952	*Mus musculus* ribosomal protein S2
TRIOBP	NM_007032	*Mus musculus* TRIO and F-actin binding protein

Supplementary Table S2

Table S2a Oligoes used in the identification of mouse genotype

Oligo name	Sequence (5'to 3')	Description
Trim59-WGF3	GCATTACAAATTAGGGATTTTGTCG	for genotype
Trim59-WGR3	CCTCAAAATTGTGCATTTCCTGTC	
ES-1R3	AAGGGTTATTGAATATGATCGGA	

Table S2b Primers used in cloning Trim59 full-length and mutants

Oligo name	Sequence (5'to 3')	Description
Trim59 FL-F	GGGGTACCCATGCACAATTTTGAGGAAGAG	For cloning Trim59 full-length
Trim59 FL-R	CGGGATCCCGTCAATGGGAAACTATTTTCCA	
Trim59 T2-F	GGGGTACCCGAAATTGCTCCAACTGGCA	For cloning Trim59 RING domain deleted fragment
Trim59 T2-R	CGGGATCCCGTCAATGGGAAACTATTTTCCA	
Trim59 T3-F	GGGGTACCCGATGATGTACGCCAGCATG	For cloning Trim59 RING, B-box, and CC domains deleted fragment
Trim59 T3-R	CGGGATCCCGTCAATGGGAAACTATTTTCCA	
Trim59 T4-F	GGGGTACCCATGCACAATTTTGAGGAAGAG	For cloning Trim59 TM domain deleted fragment
Trim59 T4-R	CGGGATCCCGTCACTTTTCATCCTTACCAGGCC	
Trim59 T5-F	GGGGTACCCATGCACAATTTTGAGGAAGAG	For cloning Trim59 B-box domain deleted fragment
Trim59 T5-R	CGGGATCCCGTCAATGGGAAACTATTTTCCA	

Table S2c Oligoes used in RT-PCR

Oligo name	Sequence (5'to 3')	Description
Trim59-F	ATGCACAATTTTGAGGAGGAG	Mouse Trim59 detection
Trim59-R	TCAACGAGAAACTATTTTCC	
β-actin-F	GCCGGGACCTGACAGACTAC	Mouse β-actin detection
β-actin-R	CGGATGTCAACGTCACACTT	

Table S2d Oligoes used in qPCR

Oligo name	Sequence (5'to 3')	Description
Trim59-F	CATATGGAGGCCTCTGCGAA	Murine Trim59 detection
Trim59-R	GGTGACAACATCTGGGTGGT	
WASH-F	CTCCCTGCTGCTCTTCAACA	Murine WASH detection
WASH-R	GACAGAGGGGCATCGAACAA	
GAPDH-F	TCAACGGCACAGTCAAGG	Murine GAPDH detection
GAPDH-R	TACTCAGCACCGGCCTCA	

Table S2e SiRNA sequences used in this study

Oligo name	Sequence (5'to 3')
Mouse Trim59	CCATGTTCCTGGTCTGATA<br>GACACACATTGGACAGATA
Mouse Trim27	CCACCTAAGAAGAGTGAAA<br>GGGCTGAAAGAATCAGGAT
Mouse WASH	GGAGGAGAAATTGTTCGAT<br>CCATCTTTACTGGCGCCTT

Table S2f Probes used in *in situ* hybridization

Oligo name	Sequence (5'to 3')	Targeted sequence
Murine Trim59-1<br>Murine Trim59-2	AGCACTGCTTGCTGCTTGTGG<br>TCCCACCTGCTAACACTGGCAC	Murine Trim59
Murine Oct4-1<br>Murine Oct4-2	GAGGTAATCCACCCCCGGCTCGG<br>TGCCCTTCTGGCGCCGGTTA	Murine Oct4
Murine T gene-1<br>Murine T gene-2	CCACCGAACGCGAACTGCGA<br>ACAAGCCACCCCCATTGGGAAT	Murine T gene
Murine BMP4-1<br>Murine BMP4-2	AGCCATGAGCTCCTGCGGGA<br>GACCAGGAGGGGCCGGAGTT	Murine BMP4
Murine Wnt3-1<br>Murine Wnt3-2	CGGGAATTTGCGGATGCGCG<br>GTCCTCGTGTTGTGGCCCCG	Murine Wnt3
Murine Cer1-1	TTGCCTCTGGGGAAGGCAGACC	Murine Cer1

Murine Cer1-2	GCAATGGTCTGGTTGAAGGGCACA	
Murine Lefty2-1<br><br>Murine Lefty2-2	TCGACTCTAGGCTCGTGTCC<br><br>GGAGACTACAGATCTATCCCCCTG	Murine Lefty2
Murine Otx2-1<br><br>Murine Otx2-2	GCCAGAATCCAGGGTGCAGGTAT<br><br>GGACCCTTCTAAGGCCCCTTCCT	Murine Otx2
Mock probe	Offered by company	
